# Supplementary material for: Use of phase plate cryo-EM reveals conformation diversity of therapeutic IgG with 50 kDa Fab fragment resolved below 6 Å
Source: Sci Rep. 2024 Jun 18;14:14079. doi: 10.1038/s41598-024-62045-8 (PMC11189423; doi:10.1038/s41598-024-62045-8)
Supplement: Supplementary file 1 — Supplementary Figures. [file 41598_2024_62045_MOESM1_ESM.pdf]

SUPPLEMENTARY INFORMATION

for

Use of phase plate cryo-EM reveals conformation diversity of therapeutic IgG with 50 kDa Fab fragment resolved below 6 Å

Hsin-Hung Lin[1,2], Chun-Hsiung Wang[1,#], Shih-Hsin Huang[1, ¥], Sam Song-Yao Lin[1], Takayuki Kato[3,§], Keiichi Namba[3], Naoki Hosogi[4], Chihong Song[5], Kazuyoshi Murata[5], Ching-Hsuan Yen[6], Tsui-Ling Hsu[6], Chi-Huey Wong[6], Yi-Min Wu[1,†], I-Ping Tu[2], Wei-Hau Chang[1,6,7,*]

[1]Institute of Chemistry, Academia Sinica, Taipei, Taiwan

[2]Institute of Statistical Science, Academia Sinica, Taipei, Taiwan

[3]Graduate School of Frontier Biosciences, Osaka University, 1-3 Yamadaoka, Suita, Osaka, Japan

[4]JEOL Ltd., 1-2 Musashino 3-chome, Akishima, Tokyo, Japan

[5]Exploratory Research Center on Life and Living Systems (ExCELLS) and National Institute for Physiological Sciences (NIPS), National Institutes of Natural Sciences, 38 Nishigonaka Myodaiji, Okazaki, Aichi, Japan

[6]Genomic Research Center, Academia Sinica, Taipei, Taiwan

[7]Institute of Physics, Academia Sinica, Taipei, Taiwan

*To whom correspondence should be addressed. Email: weihau@gate.sinica.edu.tw

Present Addresses:

[#]Academia Sinica Cryo-EM Facility, Academia Sinica, Taipei, Taiwan

[¥]Institute of Preventive Medicine, National Defense Medical Center, New Taipei City, Taiwan

[†]Cryo-EM Facility, College of Medicine, National Taiwan University, Taipei, Taiwan

[§]Institute of Protein Research, Osaka University, Suita, Osaka, Japan

This file contains 6 supplementary figures

Supplementary Figure 1

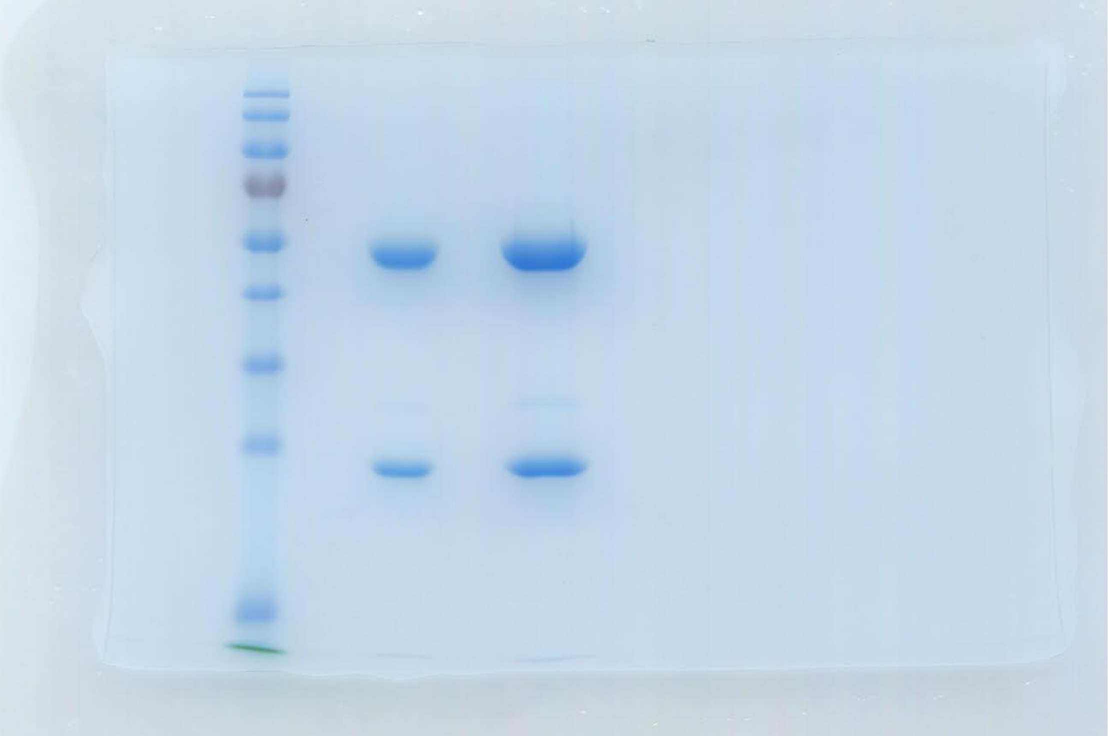

Figure S1. The original SDS-PAGE of Rituximab IgG that corresponds to Fig. 2A. Lane 1 is marker (please see annotation in Fig. 2A), lane 3 is loaded with 10 microliter IgG (5 mg/ml), and lane 5 is the same as lane 3 except that the IgG is without dilution where the concentration is kept at 10 mg/ml.

Supplementary Figure 2

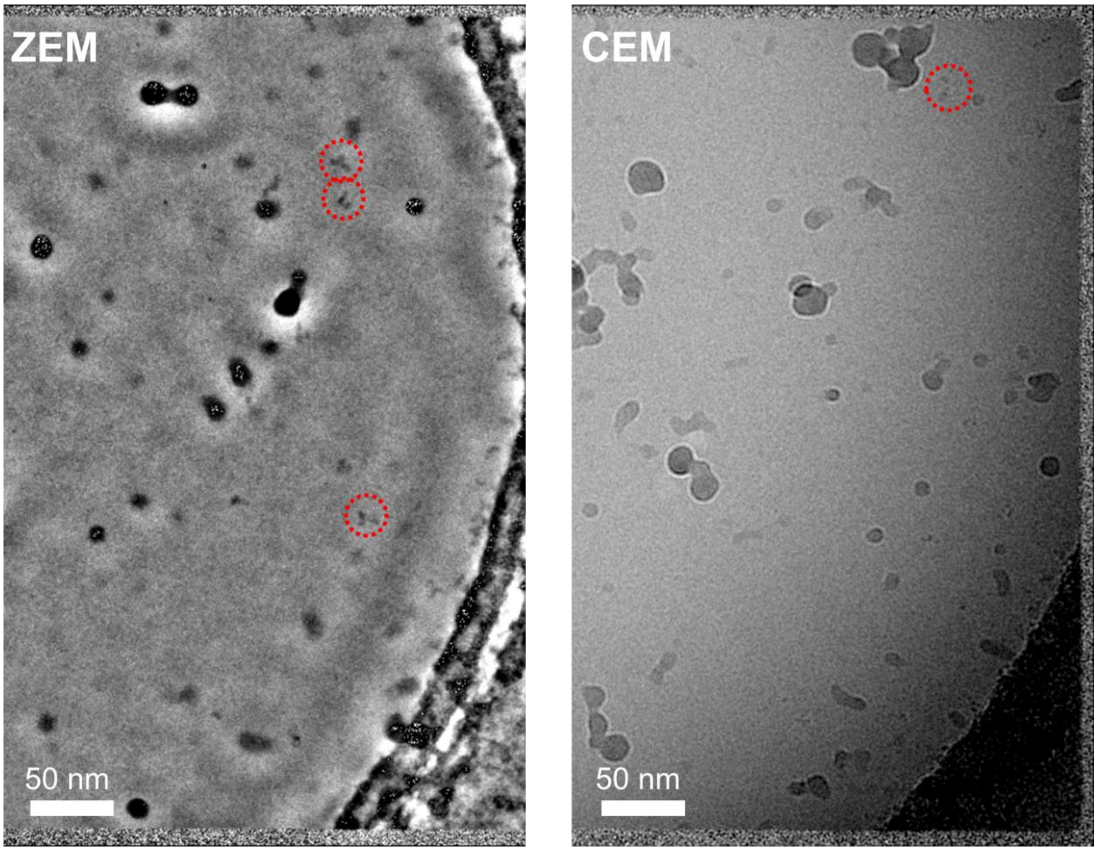

Figure S2. DDM-dispersed Rituximab IgG under phase plate and conventional cryo-EM. The left is a Zernike phase plate cryo-EM (ZEM) image of DDM-solubilized Rituximab (0.5 mg/ml with 0.008% DDM) recorded by a direct electron camera (DE-20, DDD Ltd, USA) on a FS-2200 multi-purpose 200 kV cryo-EM (JEOL Ltd, Japan) where several three-blob particles are identified and denoted by red circles. Shown in the right is a conventional cryo-EM image of the same DDM-solubilized Rituximab specimen recorded by the same camera on the same microscope where a candidate Rituximab antibody is highlighted with a red circle.

Supplementary Figure 3

A

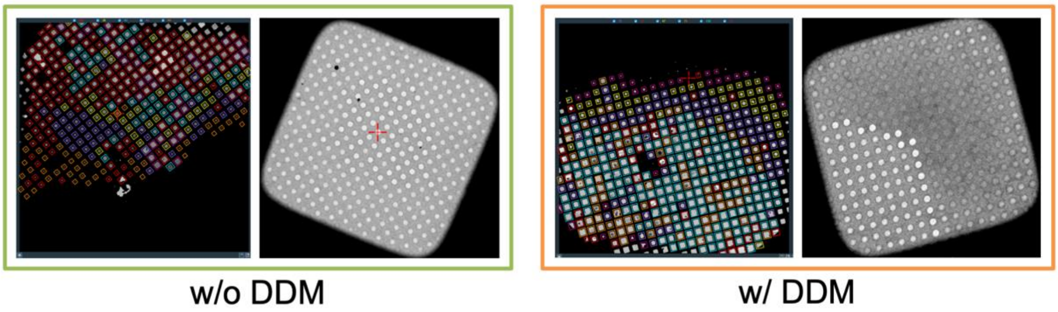

B

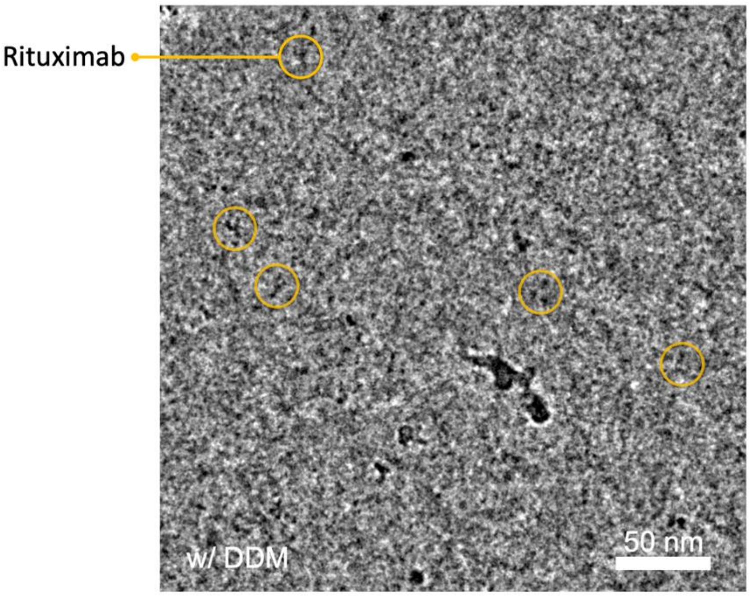

Figure S3. Low magnification atlas of entire grid with a representative grid square of two cryo-EM specimens, one without and the other with DDM (0.008%)**. (A)** Without DDM, the gray scale that reflects the ice thickness in a grid square shows the ice thickness in all holes is uniformly distributed and on the thinner side compared to that in (B). **(B)** With DDM (0.008%), the gray scales in a grid square become diversified where ~70% of holes are apparently thicker. **(C)** A high magnification micrograph of specimen of Rituximab with DDM (0.008%) was recorded by conventional cryo-EM technique with -3µm defocus value. A Rituximab molecule is circled where the background is very noisy. All images were recorded on Talos Arctica (Thermo Fisher, USA).

Supplementary Figure 4

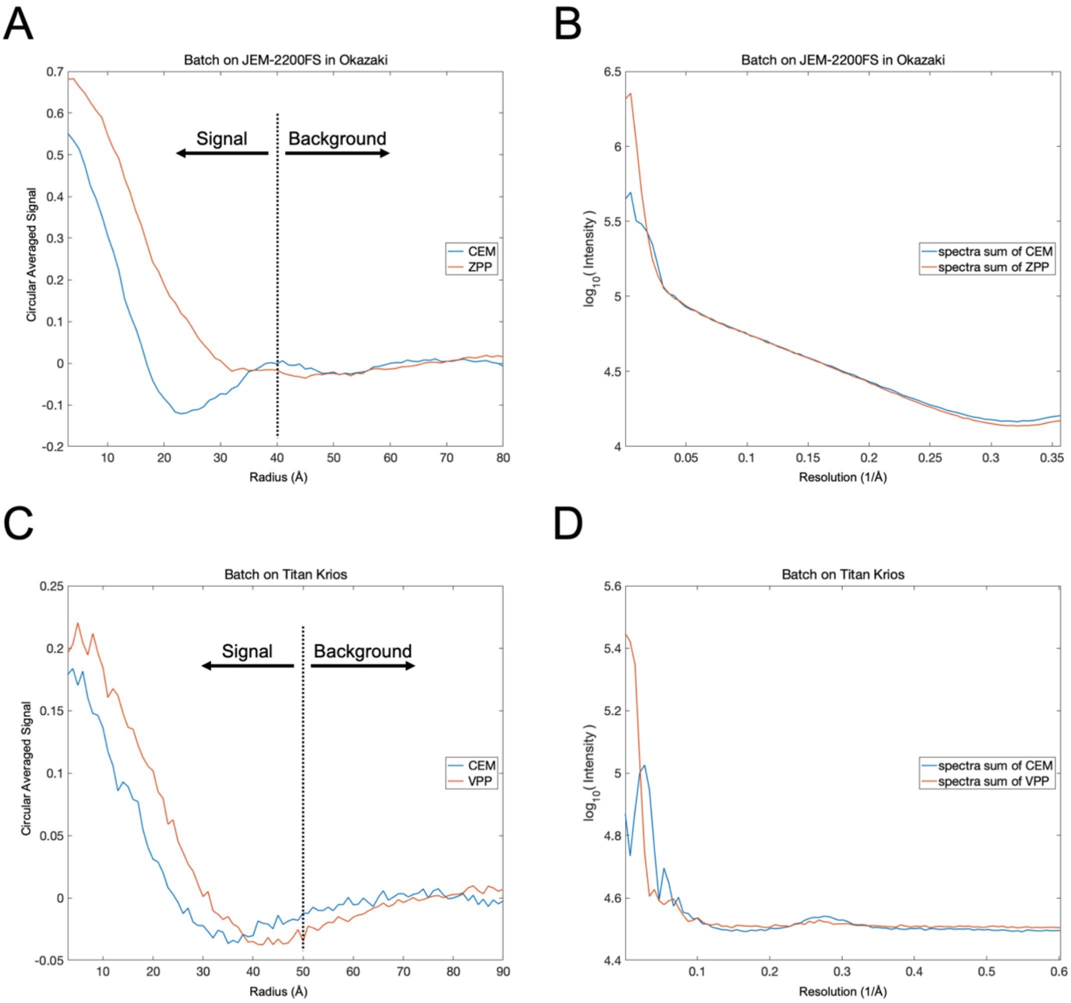

Figure S4. Contrast enhancement by phase plates characterized by signal to background in cryo-EM image and spatial frequency in Fourier transform. (A) Signal to background of ZPP Fab image (in red) and CEM Fab image (in blue). The particle profile is obtained from rotational averaging and plotted against particle radius. The images were taken by a direct electron camera (DE-20, DDD, USA) on FS-2200 200 kV multi-purpose cryo-EM (JEOL, Japan). **(B)** Comparison of ZPP and CEM Fourier transforms. ZPP transform has 5 times higher amplitude than CEM with 3 micron defocus) at very low spatial frequency. **(C)** Signal to background of VPP image (in red) and CEM image (in blue) as plotted against particle radius. The images were taken by a direct electron camera (K3, Gatan, USA) on Titan Krios 300 kV cryo-EM (Thermo Fisher, USA). **(D)** Comparison of VPP and CEM Fourier transforms shows at very low spatial frequency regime, the VPP has 3 times higher amplitude than CEM (3 micron defocus).

Supplementary Figure 5

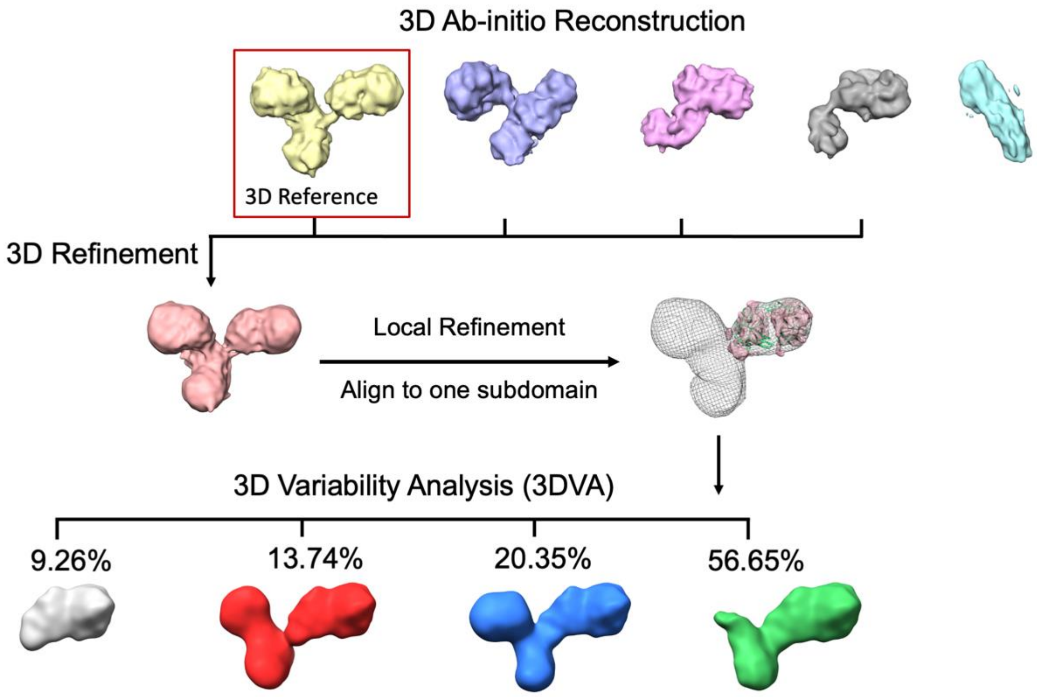

Figure S5. A workflow of 3D reconstruction for Rituximab IgG from ab initio using VPP cryo-EM images using cryoSPARC. Each toss had resulted in a different initial model, where the frequency of getting the three-arm structure is ~30%. 3D refinement were carried out in two stages, global refinement that optimized the alignment parameters of each IgG particle image, and local refinement (focused refinement) that focus on the alignment of the Fab domain by masking it in each IgG image. Further 3DVA analysis, also using cryoSparc to resolve heterogeneous conformations. A total of four meaningful conformations are obtained, two are with three arms (red and blue), and one with two arms (green), and one with one arm (grey).

Supplementary Figure 6

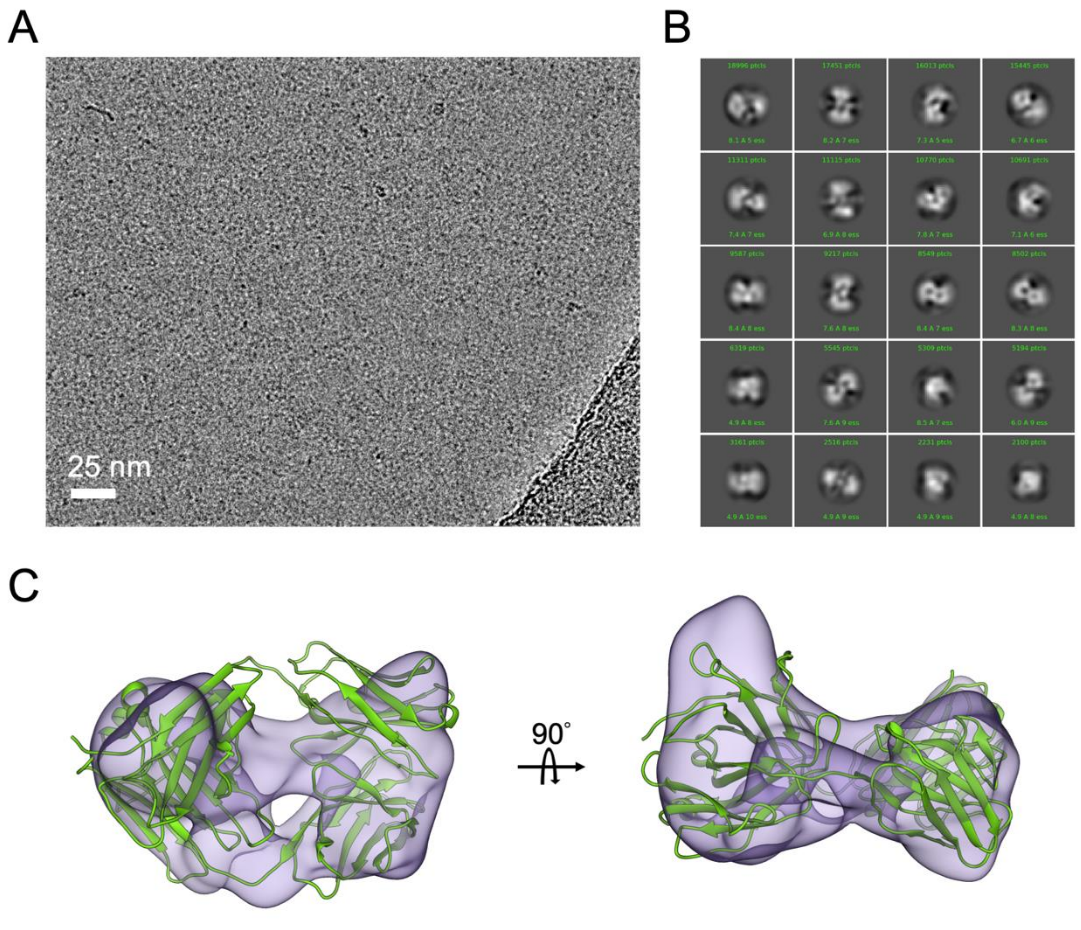

Figure S6. Conventional cryo-EM imaging of Rituximab Fab fragment. (A) A defocused CEM image shows Fab particles with highly noisy background. **(B)** Representative 2D class averages. **(C)** 3D reconstruction of Fab fragment with docking of X-ray model (PDB:4KAQ).
